# Rapid assessment of knowledge, attitudes, practices, and risk perception related to the prevention and control of Ebola virus disease in three communities of Sierra Leone

**DOI:** 10.1186/s40249-016-0142-9

**Published:** 2016-06-06

**Authors:** Hai Jiang, Guo-Qing Shi, Wen-Xiao Tu, Can-Jun Zheng, Xue-Hui Lai, Xin-Xu Li, Qiang Wei, Mei Li, Li-Quan Deng, Xiang Huo, Ming-Quan Chen, Feng Xu, Long-Jie Ye, Xi-Chen Bai, Tong-Nian Chen, Shao-Hua Yin, Thomas T. Samba, Xiao-Feng Liang

**Affiliations:** State Key Laboratory for Infectious Disease Prevention and Control, Collaborative Innovation Center for Diagnosis and Treatment of Infectious Diseases, National Institute for Communicable Disease Control and Prevention, Beijing, 102206 China; Chinese Center for Disease Control and Prevention, Beijing, 102206 China; ITERP, Beijing, 102206 China; District Health Management Team, Western Area, Ministry of Health and Sanitation, Freetown, Sierra Leone

**Keywords:** Knowledge, Attitude, Practice, Risk perception, ITERP, Ebola, Sierra Leone

## Abstract

**Background:**

The recent outbreak of the Ebola virus disease (EVD) in Sierra Leone has been characterized by the World Health Organization as one of the most challenging EVD outbreaks to date. The first confirmed case in Sierra Leone was a young woman who was admitted to a government hospital in Kenema following a miscarriage on 24 May 2014. On 5 January 2015, intensified training for an EVD response project was initiated at the medical university of Sierra Leone in Jui. To understand the knowledge, attitudes, practices, and perceived risk of EVD among the public, especially after this training, a rapid assessment was conducted from 10 to 16 March 2015.

**Methods:**

Interviews were conducted with 466 participants based on questionnaires that were distributed from 10 to 16 March 2015 by cluster sampling in three adjacent communities, namely Jui, Grafton, and Kossoh Town, in the Western Area Rural District of Sierra Leone.

**Results:**

It was found that knowledge about EVD was comprehensive and high. Positive attitude towards prevention was found to be satisfactory. Nearly all participants knew the reporting phone number 117 and had reported some change in behavior since learning about Ebola. More than half (62 %) of the participants had a history of travelling to urban areas, which increases the risk of infection. The multivariable logistic regression analysis showed that community and occupation were variables associated with perceived risk of EVD.

**Conclusions:**

Our study showed that community level social mobilization and community engagement were an effective strategy in the special context.

**Electronic supplementary material:**

The online version of this article (doi:10.1186/s40249-016-0142-9) contains supplementary material, which is available to authorized users.

## Multilingual abstracts

Please see Additional file 1 for translations of the abstract into the six official working languages of the United Nations.

## Background

The recent outbreak of Ebola virus disease (EVD) in Sierra Leone has been characterized by the World Health Organization as one of the most challenging EVD outbreaks to date [1, 2]. The first confirmed case in Sierra Leone was a young woman who was admitted to a government hospital in Kenema following a miscarriage on 24 May 2014 [3] There have been 8 590 cumulative cases attributed to EVD as of 30 April 2015from WHO [4]. The Western Area Rural District of Sierra Leone is a main area of interest with high risk of Ebola transmission [5].

On 5 January 2015, intensified training for an EVD response project (ITERP) was initiated at the medical university of Sierra Leone in Jui. The project’s overall goal is to help the three administrative villages (Jui, Grafton and Kossoh Town) to strengthen the capacity of EVD control, improve the network for the prevention and treatment of infectious diseases, effectively control the spread of EVD in the community, and explore sustainable infectious disease prevention and control strategies and measures. Village leaders, community leaders, religious leaders, and community volunteers from some local and administrative villages have been trained to improve the public’s awareness in order to change behaviors towards EVD control. The ITERP was undertaken in three main wards, namely Grafton, Kossoh Town, and Jui, where 42 749 people lived in 9 406 households, as of early 2015 [6].

Strategies for reducing Ebola cases to zero include detecting Ebola cases in a timely fashion, completely tracing and managing contacts, implementing safe and dignified burials, and the prevention and control of infections [7]. The first step is to engage social mobilization by involving community leaders and activists that can help improve the public’s awareness, attitudes, and practices towards EVD control [8]. The district health management team of the Western Area Rural District and the public health team from China continue to place a major focus on educating the public on how to prevent the transmission of EVD, as well as encouraging people to promptly seek medical care in the event that they experience signs and symptoms associated with the disease. Despite these efforts, public education and social mobilization campaigns have been met with varied resistance from communities.

During ITERP, it was found that some people still engage in poor behaviors, including an unwillingness to report Ebola, a preference for traditional healing, and unsafe burials. Willingness to report Ebola needs to be further strengthened in communities. To grasp community knowledge, attitudes, and behavior, especially in terms of reporting awareness, reporting phone numbers, and risk perception related to Ebola control and to identify barriers that hinder the containment of the EVD epidemic, 30 social mobilizers from Jui, Grafton, and Kossoh Town conducted a questionnaire survey with the public.

## Methods

House to house survey (Additional file 2) was conducted by 30 trained social mobilizers, separated into the three communities from 10 to 16 March 2015. A cluster sampling design was used in the study. The survey sample comprised all 35 villages in Jui (13), Kossoh Town (5), and Grafton (17). Random sampling was used to select 466 households for interviews. The household selection procedure in each village was as follows: A) Select 3–4 households in the north, south, east, and west of each village. Randomly select the first household in each direction and then choose the next-door neighbor, and so on. Members from at least 12 households were successfully interviewed in each village. B) Randomly select one eligible family member (between the ages of 20 and 60) from each selected household. C) Make sure to record participants’ valid phone numbers on the questionnaire.

About 5 % of questionnaires were checked for the quality of assessment. Risk perceptions were operationalized by asking participants whether they were at risk of contracting EVD and how they perceive their personal probability of acquiring EVD on three levels: high, medium, and low. Risk perceptions were evaluated in the following scenarios: at work, in public places and at communities. “Perceived personal probability of infection” describes participants’ estimation of the actual risk for infection.

To test the differences among the variables, we used the *χ*2 test for categorical variables (community, occupation, education level, ever been to seaside, getting Ebola information from billboards, and getting Ebola information from brochures)relating to concern about EVD. In addition, we performed explorative multivariable logistic regression analyses to assess the risk perception and sociodemographic factors associated with concern about EVD. Analyses were performed using the SPSS software version 20.0 (IBM, USA).

This study was approved by the Ethics Committee of the district health management team of the Western Area Rural District of Sierra Leone. All participants gave written informed consent before participating in the study.

## Results

### Sociodemographic characteristics

We interviewed 466 participants in the three communities. Slightly more than half were female and one third was businessperson. 27.68 % was junior high school student and 55.37 % was Muslim (see Table 1).

**Table 1:** Sociodemographic characteristics of 466 participants in three communities of Sierra Leone, 2015

Variable	*N* (%)
Community	
Kossoh town	79(16.95)
Jui	160(34.33)
Grafton	227(48.71)
Sex	
Male	224(48.07)
Female	242(51.93)
Occupation	
Student	23(4.93)
Teacher	35(7.51)
Company staff	37(7.94)
Government staff	62(13.30)
Other	71(15.24)
Petty trading	97(20.81)
Businessperson	147(31.54)
Education	
Primary school	62(13.30)
High school	74(15.88)
Illiterate	98(21.03)
University	103(22.10)
Junior high school	139(27.68)
Religion	
Christian	208(44.63)
Muslim	258(55.37)

### Rate of awareness of Ebola signs and symptoms, transmission routes, and control measures

**Fig. 1:**
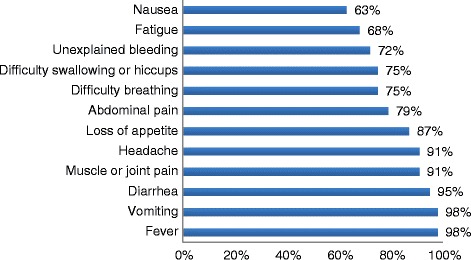
Rate of awareness of Ebola signs and symptoms

With regards to symptoms, 98 % of respondents knew that fever and vomiting are main symptoms of Ebola infections, 95 % knew that diarrhea is a main symptom, and 72 % knew that hemorrhage is also a main symptom (see Fig. 1).

**Fig. 2:**
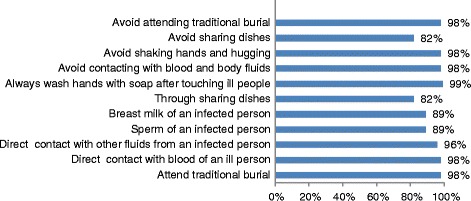
Rate of awareness of Ebola transmission routes and control measures

As for transmission routes, 98 % of the respondents thought that taking part in a traditional funeral is a main route of transmission. Other routes of transmission such as patient semen and breast milk as well as eating with patients, was known by 89 %, 89 %, and 82 %, respectively. Nearly everyone (98–99 %) reported some change in behavior, such as avoid contacting with blood and body fluids, and avoid attending traditional burial and so on, since learning about Ebola (see Fig. 2).

### Ebola infections and related awareness

**Fig. 3:**
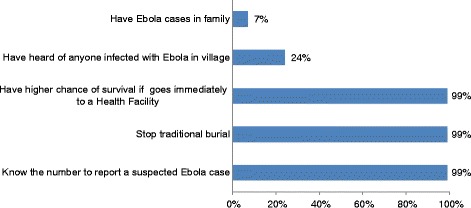
Ebola infections and related awareness

Out of the respondents, 7 % reported to have an Ebola patient in their home and 24 % had heard of Ebola infections in their village. Nearly everyone knew the reporting phone number 117 and believes that a person with Ebola has a higher chance of survival if he/she gets treatment early (99 %). Nearly everyone said that they would call the number immediately if they found out that a neighbor was going to perform a traditional burial (99 %) (see Fig. 3).

### Risk perception

Only a small number (10 %) of respondents believe that they are at not at risk of contracting Ebola because they strictly abide by medical rules. Among 90 % of respondents with infection risk, high, medium, and low risk of infection ratios were 27 %, 29 %, and 44 %, respectively. The multivariable logistic regression analysis showed that community and occupation were more likely to be associated with Ebola risk perception than the other variables (see Table 2).

**Table 2:** Association between the variables of community and occupation, and risk perception^a^

Characteristics	Odds ratios	(95 % *CI*)	*P*-value
Community			
Jui	0.29	(0.19,0.44)	<.001
Kossoh Town	0.05	(0.03,0.12)	<.001
Grafton	Ref		
Occupation			
Government staff	0.62	(0.32,1.17)	0.139
Company staff	1.30	(0.61,2.77)	0.497
Petty trading	3.18	(1.83,5.54)	<.001
Teacher	1.25	(0.51,3.03)	0.624
Student	0.61	(0.26,1.46)	0.271
Other	0.94	(0.54,1.64)	0.829
Businessperson	Ref		

^a^Multivariable logistic regression done by ordinal regression on SPSS 20.0 using the logit method

### Mobility of the population

**Fig. 4:**
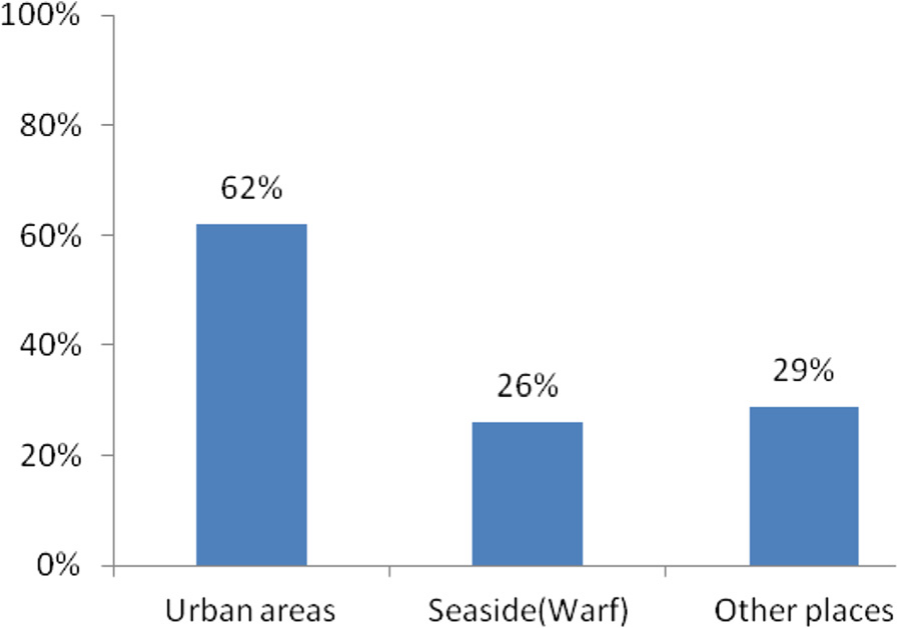
Mobility of the population in last three months

**Fig. 5:**
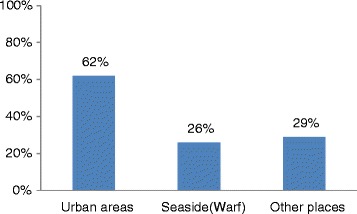
Frequency of going to urban areas

The population is mobile: 62 % (291) visited urban areas in the last three months and 25 % visited daily on average, (see Figs. 4 and 5).

### Primary channel of receiving information about EVD

**Fig. 6:**
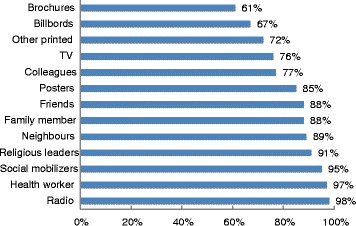
Primary channel of receiving information about EVD

Almost all respondents (98 %) prefer getting EVD-related information through the radio, making it the most favored method. Brochures are not available easily in remote villages, making it an unpopular method of information dissemination (see Fig. 6).

## Discussion

As large-scale community-based education on Ebola prevention was conducted, knowledge about EVD was found to be comprehensive and high in the communities studied, and the positive attitude towards prevention was found to be satisfactory. Comprehensive knowledge is a critical component for increasing the likelihood of adopting promoted prevention strategies and medical-seeking behaviors. As for mobility, 62 % of participants had a history of travelling to urban areas, which may increase the risk of infection.

Before this investigation, a baseline survey conducted on Ebola awareness showed that the rates of awareness of symptoms and transmission routes were 76 % and 82 %, respectively (unpublished). During ITERP, the training effectiveness was obvious. The training increased awareness of EVD control and prevention, as well as community engagement. It also established a mechanism for coordination and cooperation between the community and a professional team.

As expected, awareness of knowledge, attitudes, and practices related to EVD prevention and control among the participants was very high. This response might be attributable to the fact that individuals gained key information through the intensified education and mobilization [9]. For example, nearly everyone knew the number to call to report a suspected EVD case and to stop a traditional burial. Nearly everyone also reported some change in behavior since learning about Ebola.

The percentage of people reporting that they wash their hands with soap after touching ill people is high (99 %) and the percentage of people reporting that they avoid physical contact is also high (98 %). The reason why some residences do not report and/or still partake in unsafe burial practices seems to be related to risk perceptions. Those who felt that they were not at risk were unwilling to take action to change their behaviors. Reported risk perceptions are related to community and occupation. If real Ebola cases are reported in a community, people may have a higher perception of the actual risk for infection. The differences between occupations can be understood because higher mobility through urban areas and villages enhanced the infection risk For future campaigns, community education should be emphasized, not only to let people know how to prevent illness, but also why to prevent it, and to demonstrate the existence of risks [10].

During ITERP, the Chinese public health training team strengthened health education, community mobilization, case detection and management, and close contact tracing. Through multiple stages of intensive training, personnel received a significant boost. After this survey, a third public health and training team launched a big round of 10 000 brochures distribution activities, which will provide an effective weapon to win the battle for zero Ebola cases.

This study had some limitations. Restricted regional data collection might not represent the perceptions of the general population in the Western Area Rural District. Furthermore, because the investigators were social mobilizers, their level of knowledge of EVD might have affected the perceptions of the interviewees. The design of the sample size did not take into account the need for a multifactor analysis, so the results might be biased.

## Conclusion

In summary, knowledge about EVD was found to be comprehensive and high in this study. Positive attitude towards prevention was found to be satisfactory. However, it is still urgent to further strengthen public education on the symptoms and modes of transmission of Ebola in all villages. Our study showed that community level social mobilization and community engagement were an effective strategy in the special context.

## Additional files

Additional file 1: Multilingual abstracts in the sex official working languages of the United Nations. (PDF 246 kb) Additional file 2: Questionnaire on Ebola knowledge, attitudes, practices, and risk perception in three communities of Sierra Leone, 2015. (DOCX 34 kb)

## Abbreviations

EVD: Ebola virus disease
ITERP: intensified training for an EVD response project
