# Supplementary material for: Rapid assessment of knowledge, attitudes, practices, and risk perception related to the prevention and control of Ebola virus disease in three communities of Sierra Leone
Source: Infect Dis Poverty. 2016 Jun 6;5:53. doi: 10.1186/s40249-016-0142-9 (PMC4893841; doi:10.1186/s40249-016-0142-9)
Supplement: Additional file 1: — Multilingual abstracts in the sex official working languages of the United Nations. (PDF 246 kb) [file 40249_2016_142_MOESM1_ESM.pdf]

Translation of the abstract into the six official working languages of the United Nations

التقييم السريع للمعرفة والمواقف والممارسات وإدراك المخاطر المتعلقة بالوقاية من فيروس إيبولا والسيطرة عليه في ثلاث مناطق من سيراليون

هاي جيانغ، غو- كنغ شي، وان – إكساو تو، كان – جون زهنغ، شيويه هوى لاي، شين شو لي، كيانغ واي، مي لي، لي – كوان دانغ، تشانغ هيو، مينغ – كوان تشن، فينغ تشو، لونغ – جي يا، تشي – شان باي، تونغ – نيان شان، شاو – هوا يين، الدكتور طوماس تي سامبا، تشاو – فينغ ليانغ

ملخّص

تقديم عام: صنفت منظمة الصحة العالمية مؤخرا انتشار الإيبولا كأكثر الأمراض تفشيًا إلى اليوم وقد تم تسجيل أول حالة إصابة مؤكدة في سيراليون والتي أصابت امرأة شابة حيث تم نقلها إلى المستشفى الحكومي في كينيما بعد أن أجهضت يوم 24 مايو 2014. في 5 يناير 2015 بدأ التدريب المكثّف لمشروع الاستجابة لفيروس إيبولا بالجامعة الطبية في سيراليون بمنطقة جويي بهدف فهم المعرفة والمواقف والممارسات وإدراك المخاطر المتعلقة بفيروس إيبولا بين الناس وبعد هذا التدريب أجري تقييم سريع من 10 إلى 16 مارس 2015.

التقنيات المستخدمة: أجريت مقابلات مع 466 مشارك اعتمادا على الاستبيانات التي وُزعت من 10 إلى 16 مارس 2015 استنادا على 3 مجتمعات لتمثل 3 مناطق مجاورة: جويي وغرافتون و كوسو في المنطقة الريفية الغربية في سيراليون.

النتائج: تبين أن المعرفة المتعلقة بفيروس إيبولا هي معرفة شاملة وثريّة وقد تم العثور على طريقة ايجابية ومُقنعة للوقاية منه. إنّ جميع المشاركين تقريبا على دراية برقم هاتف الإبلاغ 117 وقد تم ملاحظة بعض التغيير في السلوك منذ أن توسعت المعرفة حول فيروس إيبولا. لقد كان أكثر من نصف عدد المشاركين (62%) يتنقلون إلى المناطق الحضرية وهو ما يزيد من ارتفاع خطر العدوى. أظهر تحليل الانحدار اللوجستي متعدد المتغيرات (multivariable logistic regression analysis) أن انتشار المرض في المجتمعات يتغيّر حسب إدراك أفراده بمخاطر هذا الفيروس.

الخاتمة:أظهرت دراستنا أن التعبئة الاجتماعية على مستوى المجتمع إضافة إلى إشراك المجتمع المحلي تُعتبر استراتيجيةً فعّالة في سياق خاص.

Translated from English version into Arabic by Zeineb Trabelsi, through

TRANSLATORS WITHOUT BORDERS

塞拉利昂三个社区埃博拉防控知识、态度、行为和风险感知快速评估

姜海，施国庆，涂文校，郑灿军，来学惠，李新旭，魏强，李美，邓立权，霍翔，陈明泉，徐峰，叶龙杰，白熙臣，陈同年，殷邵华，Thomas T. Samba，梁晓峰

摘要

引言：发生在塞拉利昂的埃博拉出血热疫情被世界卫生组织视为最具有挑战性的一次全球公共卫生事件。2014 年 5 月 24 日，来自塞拉利昂凯内马政府医院的一名流产女性被确认为首例埃博拉出血热病例。为进一步巩固和扩大我国援助塞拉利昂公共卫生师资培训效果，有效阻断埃博拉出血热的社区传播，我国援助塞拉利昂公共卫生师资培训队启动实施了在塞拉利昂西区农村地区的三个社区的埃博拉出血热重点培训项目（Intensified Training for Ebola Response Project, 简称 ITERP），项目时间为 2015 年 1 月 5 日～4 月 19 日，共 15 周。为更好地推进中国-塞拉利昂三个示范区重点培训项目工作，掌握示范区人群关于埃博拉防控知识、态度、行为，特别是报病意识和报病电话的知晓程度，以及人群风险感知情况，为完善和加强埃博拉健康教育、社区动员提供依据。中国现场流行病学培训项目成员于 2015 年 3 月 10-16 日在弗里敦地区 Jui、Grafton 和 Kossoh Town 三个示范区开展了本次调查。

方法：根据调查问卷，调查员对塞拉利昂西区农村地区 Jui（13 个村）、Grafton（5 个村）和 Kossoh Town（12 个村）三个示范区共 30 个村进行整群抽样，然后每个村各随机抽取 14 户，每户随机调查一名（年龄为 20-60 岁），累计 466 户。

结果：被调查者埃博拉出血热防控的综合常识知晓率很高。报病电话117知晓率为99.13%；培训后行为也有相应的改变；62%的被调查者最近三个月有外出城区史，增加了感染风险。多因素回归分析表明不同社区和职业的的居民风险感知不同。

结论：经过多阶段强化培训，参加培训人员的埃博拉知识和防控理念得到显著提升，认识到在资源有限的

情况下仍然可以通过发动群众、依靠群众有效控制埃博拉出血热。

Translated from English version into Chinese by Hai Jiang

Tour d'horizon des connaissances, attitudes, pratiques et perceptions des risques associées à la prévention et à la lutte contre la fièvre d'Ébola dans trois localités du Sierra Leone

Hai Jiang, Guo-Qing Shi, Wen-Xiao Tu, Can-Jun Zheng, Xue-Hui Lai, Xin-Xu Li, Qiang Wei, Mei Li, Li-Quan Deng, Xiang Huo, Ming-Quan Chen, Feng Xu, Long-Jie Ye, Xi-Chen Bai, Tong-Nian Chen, Shao-Hua Yin, Thomas T. Samba, Xiao-Feng Liang

Résumé

Contexte: Selon l'Organisation mondiale de la Santé, la récente épidémie de fièvre d'Ébola au Sierra Leone a été l'une des plus difficiles à endiguer jusqu'à présent. Le premier cas confirmé était une jeune femme, hospitalisée à Kenema le 24 mai 2014 à la suite d'une fausse couche. Le 5 janvier 2015, une formation intensive dans le cadre d'un projet de réponse à l'épidémie d'Ébola était mise en place à la faculté de médecine du Sierra Leone à Jui. Une évaluation rapide a été menée du 10 au 16 mars 2015 afin de comprendre les connaissances, attitudes, pratiques et perceptions des risques liés à la fièvre d'Ébola dans le grand public, en particulier après cette formation.
Méthodes: Des entretiens ont été menés auprès de 466 participants au moyen de questionnaires distribués entre le 10 et le 16 janvier, par échantillonnage en grappes, dans les trois localités voisines de Jui, Grafton et Kossoh Town, dans le district rural de l'Ouest du Sierra Leone.
Résultats: Il s'est avéré que les connaissances relatives à la fièvre d'Ébola étaient bonnes et complètes. L'attitude vis-à-vis de la prévention était positive et satisfaisante. Presque tous les participants connaissaient le numéro à appeler pour signaler un cas (le 117) et signalaient avoir changé certains comportements depuis qu'ils avaient acquis des connaissances sur l'infection. Plus de la moitié des participants (62%) s'étaient rendus dans des zones urbaines, ce qui augmentait le risque d'infection. L'analyse de régression logistique multivariée a montré que l'appartenance communautaire et la profession étaient des variables associées au risque perçu d'infection par le virus d'Ébola.
Conclusions: Notre étude a montré que la mobilisation sociale au niveau communautaire et l'implication des populations ont constitué une stratégie efficace dans le contexte en présence.

Translated from English version into French by Suzanne Assenat, through

TRANSLATORS WITHOUT BORDERS

Краткий опрос знаний, позиций, практик и восприятия риска касающихся профилактики и контроля лихорадки Эбола в трех районах Сьерра-Леоне.

Хай Цзян, Го-Цин Ши, Вень-Сяо Ту, Цань-Цзюнь Чжен, Сюэ-Хуэй Лай, Синь-Сюй Ли, Цян Вей, Мэй Ли, Ли-Цюань Ден, Сян Хо, Мин-Цюань Чэнь, Фэн Сюй, Лун-Цзе Е, Си-Чень Бай, Тун-Нянь Чэнь, Шао-Хуа Инь, преп. Доктор Томас Т. Самба и Сяо-Фэн Лян.

Тезисы

Историческая справка: Недавняя вспышка лихорадки Эбола (EVD) в Сьерра-Леоне была охарактеризована Всемирной Организацией здравоохранения как одна из самых серьезных на сегодняшний день. Первым подтвержденным случаем в Сьерра-Леоне была молодая женщина, поступившая в правительственную клинику в Кенеме 24 мая 2014 года после выкидыша. 5 января 2015 года в медицинском университете Джуи было запущено интенсивное обучение по проекту реагирования на EVD. Для понимания информации, позиций, практик и восприятия риска EVD среди населеия, особенно после этого обучения, с 10 по 16 марта 2016 года был проведен краткий опрос.
Методы: Опрос был проведен среди 466 участников, основываясь на списке вопросов, распространенных с 10 по 16 марта 2016 года выборочно группами в трех прилегающих населенных пунктах, а именно: Джуи, Графтон и Коссо таун в сельском дистрикте Западной области Сьерра-Леоне.
Результаты: Было выявлено, что знания о вирусе Эбола бостаточно осознаны и значительны. Распространено положительное отношение к профилактике заболевания. Почти все респонденты знакомы с номером телефона 117, по которому необходимо заявить о случае заболевания и отметили изменение в поведении после образовательной кампании. Более половины опрошенных (62%) сообщили о своих поездках в городскую зону, что увеличивает риск инфицирования. Многоаспектный логистический регрессивный анализ показал, что населенный пункт и основное занятие являются аспектами, связанными с восприятием риска лихорадки Эбола.
Выводы: Наше исследование показало, что социальная мобилизация на коллективном уровне и участие общины являются эффективной стратегией в особом контексте.

Translated from English version into Russian by Anna Haas, through

TRANSLATORS
WITHOUT BORDERS

Evaluación rápida de conocimientos, actitudes, prácticas y percepción de riesgo en la prevención y control de la enfermedad por el virus del ébola en tres comunidades de Sierra Leona

Hai Jiang, Guo-Qing Shi, Wen-Xiao Tu, Can-Jun Zheng, Xue-Hui Lai, Xin-Xu Li, Qiang Wei, Mei Li, Li-Quan Deng, Xiang Huo, Ming-Quan Chen, Feng Xu, Long-Jie Ye, Xi-Chen Bai, Tong-Nian Chen, Shao-Hua Yin, Rev. Dr. Thomas T. Samba, y Xiao-Feng Liang

Resumen

Antecedentes: El brote reciente de la enfermedad por el virus del ébola (EVE) en Sierra Leona ha sido caracterizado por la Organización Mundial de la Salud como uno de los brotes de EVE más desafiantes de la historia. El primer caso confirmado en Sierra Leona fue una mujer joven que fue internada en un hospital estatal de Kenema luego de un aborto espontáneo el 24 de mayo de 2014. El 5 de enero de 2015, en la universidad de medicina de Sierra Leona en Jui se inició un proyecto de entrenamiento intensivo para responder a la EVE. Para comprender los conocimientos, actitudes, prácticas y percepción de riesgo de la EVE entre el público, en especial luego de este entrenamiento, se llevó a cabo entre el 10 y el 16 de marzo de 2015 una evaluación rápida.
Métodos: Se llevaron a cabo entrevistas con 466 participantes mediante cuestionarios distribuidos entre el 10 y el 16 de marzo de 2015 por muestreo por conglomerados en tres comunidades adyacentes, a saber Jui, Grafton y Kossoh Town, en la zona oeste del distrito rural de Sierra Leona.
Resultados: Se encontró que el conocimiento sobre la EVE era extenso y elevado. Se observó que la actitud

positiva frente a la prevención era satisfactoria. Casi todos los participantes conocían el número para reportar los casos, 117, y habían reportado algunos cambios en el comportamiento desde su aprendizaje sobre el ébola. Más de la mitad (62%) de los participantes habían viajado a zonas urbanas, lo que aumenta el riesgo de infección. El análisis de regresión logística multivariante mostró que la comunidad y la ocupación eran variables asociadas con una percepción de riesgo de EVE.
Conclusiones: Nuestro estudio mostró que la movilización social a nivel comunitario y la participación de la comunidad eran estrategias efectivas en el contexto espacial.

Translated from English version into Spanish by Maria Alejandra Aguada, through

TRANSLATORS
WITHOUT BORDERS
