# Supplementary material for: Rapid assessment of knowledge, attitudes, practices, and risk perception related to the prevention and control of Ebola virus disease in three communities of Sierra Leone
Source: Infect Dis Poverty. 2016 Jun 6;5:53. doi: 10.1186/s40249-016-0142-9 (PMC4893841; doi:10.1186/s40249-016-0142-9)
Supplement: Additional file 2: — Questionnaire on Ebola knowledge, attitudes, practices, and risk perception in three communities of Sierra Leone, 2015. (DOCX 34 kb) [file 40249_2016_142_MOESM2_ESM.docx]

**Rapid Assessment on Knowledge, Attitudes, Practices and Risk Perception about EVD in 3 Communities, in Sierra Leone**

**Interviewer information and investigated Place:**

Name and phone number of social mobilizer: __________________________________

Date of investigation: __________________

Community: ① Jui ② Kossoh Town ③ Graffton □

Village name: _________

**Informed Consent：**

We are social mobilizer for combating Ebola. Now, we are going to ask you some questions about your knowledge, attitudes and practices about Ebola. Your answer will be very important for us to improve health education and social mobilization. We are working together to fight Ebola.

Do you agree to take part in this interview? ① Yes ② No □

If no, why?

**1. Name of interviewee (one person between ages of 20 and 60):**

**2. Age: (Years)**

**3. Sex:**  ① Male ② Female □

**4. If you suspect you, or your family member with Ebola, would you like to report?**

① Yes ② No □

If yes, how do you report?

And do you know the number to call?

If no, why

**5. Would you like to call immediately when you find your neighbor is going to do traditional burial?**  ① Yes ② No □

**6. Do you believe that the person with Ebola has higher chance of survival if he/she got treatment earlier?** ① Yes ② No □

**7. Have you heard of anyone infected with Ebola in your village recently?**

① Yes ② No □

**8. Were there Ebola cases in your family last year?**

① Yes ② No □

**9. Do you think that there is a chance for you to get EBOLA?**

① Yes ② No □

If yes, what is the possibility of you getting EBOLA?

① Higher ② Medium ③ Lower □

If no, why?

**10. Please fill in the following table about your activities going out of the village.**

| Going out of the village in recent 3 months | | | | | |
| --- | --- | --- | --- | --- | --- |
| Urban areas | | Seaside or Wharf | | Other places  (specify) | |
| ①Yes ②No | How often# | ①Yes  ②No | How often# | ①Yes  ②No | How often# |
|  |  |  |  |  |  |

**# Note:** ①every day on average ②every week on average

③every month on average ④almost not

**11. How many persons in your family?**

**12. How much income in your family per month on average?**  Le

**13. What is your job or what do you do for living (Occupation)?**  □

① government employee ② company employee ③ Business ④ petty trading

⑤ Teacher ⑥ Student ⑦ Others, please specify:

**14. What is your religion?**

① Christian ② Muslim ③ Others, please specify □

**15. Did you ever go to school?**  ① Yes ② No □

If Yes, What is your Education level? ① Primary school ② Secondary school □

③ Senior school ④ College or university ⑤ Other, Please specify

**16. Is there any device in your family?**

Television ① Yes ② No □

Radio ① Yes ② No □

Mobile phone ① Yes (number? ) ② No □

**17. Do you know the sign and symptom of Ebola?**  ① Yes ② No □

**If yes, please specify:**

(Note: let the interviewee talk and Interviewer put √ in the following cell when the interviewee mention that specific sign and symptom)

| Fever |  | Diarrhea |  | Loss of appetite |  |
| --- | --- | --- | --- | --- | --- |
| Vomiting |  | Nausea |  | Muscle or joint pain |  |
| Headache |  | Fatigue |  | Unexplained bleeding |  |
| Difficulty breathing |  | Abdominal pain |  | Difficulty swallowing or hiccups |  |
| Others: | | | | | |

**18. Do you know how people get Ebola?**  ① Yes ② No □

**If yes, please specify**

| Direct contact with blood of an ill person | ① Yes ② No □ | Through sharing dishes | ①Yes ② No □ |
| --- | --- | --- | --- |
| Direct contact with other fluids from an infected person | ① Yes ② No □ | Sperm of an Infected person | ①Yes ② No □ |
| Through handshakes | ① Yes ② No □ | Breast milk of an infected person | ①Yes ② No □ |
| Attend traditional burial | ① Yes ② No □ |  |  |
| Others: | | | |

**19. Do you know how to prevent Ebola?** ① Yes ② No □

**If yes, please specify**

|  | ① **Yes**  ② **No** |  | ① **Yes**  ② **No** |
| --- | --- | --- | --- |
| Avoiding contact with blood and body fluids |  | Avoid sharing dishes |  |
| Avoid attending traditional burial |  | Avoid shaking hands and hugging |  |
| Always wash hands with soap after touching ill people |  |  |  |
| Others: | | | |

**20. What are the sources of information that you get information about Ebola?**

Radio ① Yes ② No □

Health workers ① Yes ② No □

Billbords ① Yes ② No □

Brochures ① Yes ② No □

Posters ① Yes ② No □

Other printed ① Yes ② No □

TV ① Yes ② No □

Social mobilizers ① Yes ② No □

Religious leaders ① Yes ② No □

Family member ① Yes ② No □

Friends ① Yes ② No □

Neighbours ① Yes ② No □

Colleagues ① Yes ② No □

Others, please specify:

**21. What is your Phone Number or other contact information?**

**Thank you !**
